# Transient Responses to Rapid Changes in Mean and Variance in Spiking Models

**DOI:** 10.1371/journal.pone.0003786

**Published:** 2008-11-21

**Authors:** Peyman Khorsand, Frances Chance

**Affiliations:** Department of Neurobiology and Behavior, University of California Irvine, Irvine, California, United States of America; Indiana University, United States of America

## Abstract

The mean input and variance of the total synaptic input to a neuron can vary independently, suggesting two distinct information channels. Here we examine the impact of rapidly varying signals, delivered via these two information conduits, on the temporal dynamics of neuronal firing rate responses. We examine the responses of model neurons to step functions in either the mean or the variance of the input current. Our results show that the temporal dynamics governing response onset depends on the choice of model. Specifically, the existence of a hard threshold introduces an instantaneous component into the response onset of a leaky-integrate-and-fire model that is not present in other models studied here. Other response features, for example a decaying oscillatory approach to a new steady-state firing rate, appear to be more universal among neuronal models. The decay time constant of this approach is a power-law function of noise magnitude over a wide range of input parameters. Understanding how specific model properties underlie these response features is important for understanding how neurons will respond to rapidly varying signals, as the temporal dynamics of the response onset and response decay to new steady-state determine what range of signal frequencies a population of neurons can respond to and faithfully encode.

## Introduction

Cortical neurons continuously receive input from a large number of excitatory and inhibitory synapses [1]. This synaptic bombardment persists even in the absence of sensory stimuli [2]–[4], suggesting that it is internally generated by the brain [5], [6]. Background synaptic activity introduces a high degree of variability into cortical responses, apparent in both the irregularity of cortical spike trains and also the high degree of subthreshold membrane potential fluctuation [7]–[11].

The net synaptic current to a neuron, obtained from the difference between excitatory and inhibitory components, may be quite small compared to the total level of synaptic input (the sum of these two components) if the majority of excitation is cancelled by inhibition. In this case, although the mean input current may be quite small, the variability introduced into the neuronal responses can nevertheless be large. By changing excitation and inhibition independently, the mean and variance (referred to here as “noise”) of the synaptic input current can be varied independently of each other. It should be noted that although we refer to the variance of input current as “noise”, we do not mean to imply that this signal has no useful function. In fact, one purpose of this study is to further explore the consequences of using noise, or input current variance, as a possible information conduit to the neuron.

Although the presence of noise can limit the information transmission capacity of a neuron or a neuronal population [10], noise can also have a useful function in a network. For example, uniform additive or multiplicative noise correlations in a neuronal population can improve the coding accuracy of a population of neurons, although limited-range correlations have a mixed effect on population coding accuracy [12], [13].

The effects of noise on firing rates of different integrate-and-fire model neurons have been studied extensively (for examples, see [14]–[21]). Noise can linearize the firing-rate curve by removing the discontinuity at spike threshold, dampen resonance effects [14], reducing network synchronization [22], and dynamically amplify an embedded signal through stochastic resonance [23], [24] or some of its generalizations [25].

More recently, the possibility has been raised that noise itself may represent a separate conduit of information in addition to the mean input current to a neuron [26] and the consequences of embedding information in this information channel have been studied [18], [27]–[29]. Interestingly, it was suggested [28] that the noise channel is superior to the mean current channel for the fast, faithful transmission of signals. Neuronal response dynamics, however, are strongly influenced by the dynamics of action potential generation as well as noise parameters [30], [31].

In this study we examine the temporal dynamics of neuronal responses to sudden changes in either the mean or variance (noise) of the input current. For this study, we divide the firing response into two stages, the “response onset”, essentially a measure of how quickly the model neuron's firing rate reacts to a change in input, and the “decaying response”, a measure of how quickly the firing rate stabilizes to its new steady-state after a sudden change (this division is introduced mainly for clarity of presentation, as there is no true absolute division between these two stages). We find that the temporal dynamics of the response onset may be predicted based on the underlying membrane potential distribution. For this analysis, we focus primarily on integrate-and-fire models to take advantage of their mathematical tractability, but we also examine a more biologically-realistic conductance-based model. The response onset dynamics of each model differ depending on the choice of model as well as the noise parameters (these findings are in agreement with previous findings [30], [31]). We find that the decaying response, on the other hand, is well fit by an exponentially decaying sinusoidal function for all models studied here. Interestingly, the time constant of this decay has a power-law relation with input noise over a wide range of parameters.

## Methods

In this study, we primarily examined integrate-and-fire neurons, a type of single-compartment neuron model. The dynamics of the membrane potential, V(t), is governed by
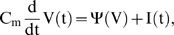
(1)where I(t) is the total input current, C_m_ is the total membrane capacitance, and Ψ(V) is a model-dependent function of membrane potential (discussed later in this section). In this study we focus on three well-known integrate-and-fire models, the leaky integrate-and-fire (LIF) model, the quadratic integrate-and-fire (QIF) model [32], [33], and the exponential integrate-and-fire (EIF) model [30].

I(t) is the sum of two components, an external component, I_ext_, analogous to an external current injected through a recording electrode, and a synaptic component, I_syn_, designed to approximate current arising from *in vivo* synaptic input [34]. I_syn_ is the sum of a Gaussian white noise process with variance, σ, and a mean current, I_m_, filtered through a linear filter with time constant τ_s_,
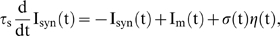
(2)where the time averages 〈η(t)〉 = 0 and 〈η(t)η(t′)〉 = δ(t−t′), and τ_s_ is the synaptic time constant. In our study, the synaptic time constant varied from 0 to 20 ms, as noted. I_m_ and σ were adjusted so that when comparing behavior of different models, the mean firing rate and decay time (the time it takes for the firing rate to reach steady-state after a change in input, see Results) were comparable across models. I_ext_ comprises the input signal, either a step in mean or variance, and does not pass through the synaptic filter (see Results).

Due to existence of the noise component in the input current, the time-dependence of an individual neuron's membrane potential is not deterministic. As a result, the membrane potential is described by a probability distribution, P(V,I,t)ΔVΔI, that describes the probability of finding the membrane potential in a range of [V,V+ΔV] when input current is in a range [I,I+ΔI] at time *t*. The probability flow vector J(V,I,t) is a measure of the net probability flux in (V,I) space. The probability distribution and probability flow are linked through a conservation/continuity equation known as the Fokker-Planck (FP) equation (see Text S1 for more details).

The FP equation connects any inhomogeneity of the probability flow, J(V,I,t), in configuration space to the change in the local probability distribution over time:
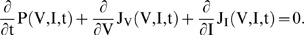
(3)In the above equation, J_V_(V,I,t) and J_I_(V,I,t) are different components of the probability flow vector. The boundary conditions imposed on the Fokker-Planck equation, as well as the Ψ(V) term, are model-dependent. For each model, the mean firing rate is equal to the total probability flow across the spike-threshold (defined by V = V_th_).

### Leaky integrate-and-fire (LIF) model

In the LIF model, Ψ_LIF_(V) is a linear function of membrane potential,
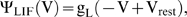
(4)where g_L_ is the membrane conductance of the model. The resting membrane potential, V_rest_ = −74 mV, sets V in the absence of any input current. If V depolarizes above a threshold potential, V_th_ = −54 mV, a spike is instantaneously generated and the membrane potential is set to the reset potential, V_reset_ = −80 mV. For large I_m_, the LIF firing rate asymptotically approaches a linear dependence on input current. In some situations, the firing rate of the LIF model can be calculated analytically [19].

### Quadratic integrate-and-fire (QIF) model

As its name indicates, Ψ_QIF_(V) depends quadratically on the membrane potential in the QIF model:
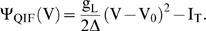
(5)I_T_ is the minimum current required to fire the neuron. Δ = V_th_−V_reset_, determines the onset of spike generation and is inversely proportional to the curvature of Ψ_QIF_(V) at its minimum, V_0_ [30]. In our simulations, V_0_ = V_rest_ to match the peak of the membrane potential probability distribution (in the subthreshold regime) to that of the LIF model. The rate of membrane potential change increases with the square of its distance from the resting potential. An action potential occurs when the membrane potential diverges to positive infinity (the dynamics of the model allow this to occur in a finite time interval), after which the membrane potential is reset to negative infinity (although see General Notes on Simulating IF Models). Other parameters were adjusted to make the steady-state firing rate curve of the QIF model similar to the LIF model. The minimum current required to drive the model to fire, I_T_ = g_L_(V_th_−V_rest_), was chosen to match the threshold current of the LIF model.

In the absence of noise (and provided that there is sufficient input current to drive the neuron), the firing rate of the model varies as the square root of the mean input current. This firing behavior matches the observed near-threshold behavior of all type I neurons. The QIF model can be mapped to the much-studied θ-models [33] with a simple transformation [35].

### Exponential integrate-and-fire (EIF) model

For the EIF model, first proposed by Fourcaud-Trocmé et al. [30], Ψ_EIF_(V) consists of a linear and an exponential term,
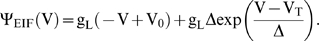
(6)As with the QIF model, the parameter Δ = (V_th_−V_reset_) is important for determining action potential onset. Its value was chosen to match the asymptotic steady-state firing rates (for large input current) of the LIF model. In the large V limit, Ψ_EIF_(V) grows superlinearly, causing V to diverge to positive infinity after sufficient depolarization. Also as the QIF model, the divergence of the membrane potential represents an action potential, but V is reset to V_reset_ after an action potential. We set V_T_ = 2V_th_−V_reset_ to match the threshold input current (in the absence of noise) to that of the LIF model. Also, V_0_ = 2V_rest_ so that in the absence of any additional current or noise, the subthreshold behavior of the EIF model is similar to the LIF model.

In contrast to the QIF model, the firing rate of the EIF is approximately linear for large input (the precise dependence is I_m_/log(I_m_)).

### General notes for simulating IF models

Model neurons were simulated using a fourth-order Runge-Kutta method. For the purposes of this study, firing rate was measured as the population firing rate of 10^5^ to 10^6^ identical neurons. I_ext_ and I_m_ were identical for each neuron in the population, but the noise component was random and different for each neuron.

When possible, we matched the parameters of the integrate-and-fire models. The membrane time constant, τ_m_ = 20 ms, and the membrane conductance, g_L_, are equal across all integrate-and-fire models. V_rest_ (for the LIF model) and V_0_ (for QIF and EIF models) were set equal to each other so that the locations of membrane-potential probability distribution peaks (in the sub-threshold regime) for different integrate-and-fire models were matched. As already noted, all other parameters were chosen to make the firing rate curves of the model as similar as possible. As a result, the models require identical threshold current for spiking, and the asymptotic dependence of firing rate on constant input current is the same for the EIF and LIF models (up to a logarithmic factor).

The membrane potential divergence (spiking mechanism) for the QIF and EIF models cannot be achieved numerically because it involves infinitely large potentials. Instead, we defined a large upper bound potential for the EIF model and a large upper and lower bound for the QIF model. The dynamics outside these boundaries, where the effect of noise is negligible, was replaced using approximate analytical expressions [30]. The firing rate is calculated by combining these numerical and analytical results.

### Conductance-Based Models

For a more biologically-realistic model, we also studied a conductance-based model proposed by Connor et al. [36]. The total membrane current, I, consists of four dynamical components in addition to I_syn_ and I_ext_:
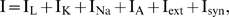
(7)

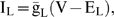


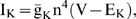


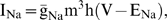


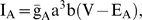
where E_L_, E_K_, E_Na_, E_A_ and g_L_, g_K_, g_Na_, g_A_ are the reversal potentials and maximal conductances of a membrane leak conductance, a delayed-rectifier potassium conductance, a fast transient sodium conductance, and a transient A-type potassium conductance, respectively. The dynamics of these conductances is described by five gating variables: n, m, h, a, and b. These gating variables, x^j^ = (n, m, h, a, b), all satisfy a simple first-order differential equation
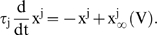
(8)More details about this model and its parameters can be found in Connor et al. [36]. In order to study the firing rate dynamics with fine time resolution, it was necessary to choose a clear-cut definition for when an action potential fires. Unless otherwise specified, we used a spike detection threshold of 20 mV. Choosing a different detection threshold did not affect our results (not shown) because of the rapidness of sodium activation.

## Results

We seek to examine the temporal dynamics of firing responses to input signals embedded in either the mean or the variance (referred to here as “noise”) of the input current. Common methods of quantitatively studying signal transmission include examining the firing-rate response of neurons to step functions in their inputs [28], [37] and measuring the modulation coefficients, first harmonics in output Fourier component of the firing rate when driven by oscillating input [18], [30], [31]. We employ the former by introducing an external injected current, I_ext_, and studying the firing responses of neuron models to steps in the mean and in the variance (noise) of I_ext_. Like I_syn_, I_ext_ is the sum of a mean current and a Gaussian white noise (see Methods). However, I_ext_ does not pass through the synaptic filter and thus is unaffected by τ_s_ (the synaptic time constant – see Methods). We chose these input signals for simplicity of analysis and also because understanding the firing responses to these inputs lays the foundation for understanding IF model responses to more complicated, fast-varying input signals.

We examine two basic features of IF model responses to steps in input signals: the “response onset” and subsequent “decaying response”. Understanding what factors modulate the “response onset” provides insight into how quickly the firing rate of a neuron or a population of neurons can react to time-varying input. Any components in the input that vary faster than the time scale of the response onset will be suppressed in the neuronal firing response. The “decaying response”, on the other hand, describes the approach of the neuronal firing rate to a new steady-state value. This response component is a measure of how quickly a network “forgets” a change in input signal. Any signal that varies at time scales slower than the population response decay time will be reflected faithfully in the population firing rate.

### Response Onset to Step Functions

#### Leaky integrate-and-fire model response onset

Because Ψ_LIF_(V) is linear for the leaky integrate-and-fire (LIF) model (see Methods), the 2-dimensional Fokker-Planck (FP) equation can be reduced to an effective 1-dimensional Fokker-Planck (FP) equation [38] for P(V,t) = ∫ dI P(V,I,t) and
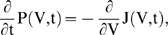
(9)where P is the probability distribution of the membrane potential (see Methods). The above equation explicitly can be written as,
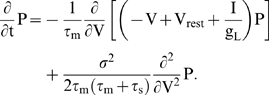
(10)where the probability vector J(V,t) = ∫ dI J_I_(V,I,t). The membrane conductance of the model is referred to as g_L_ and the membrane time constant, τ_m_. I is the mean input current to the neuron, I_m_+〈I_ext_〉. The synaptic input current consists of I_m_, the mean, and a Gaussian white noise process with variance σ^2^, filtered through a linear filter with time constant τ_s_. The LIF model mean firing rate, ν(t), in this dimensionally reduced form, is equal to boundary value J at spike threshold, J(V_th_,t). For the LIF model, the probability flow can be written as
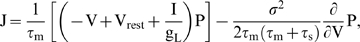
(11)where the first and the second terms on the right hand side are called the drift and diffusion terms, respectively (see Text S1 for more information).

**Figure 1:**
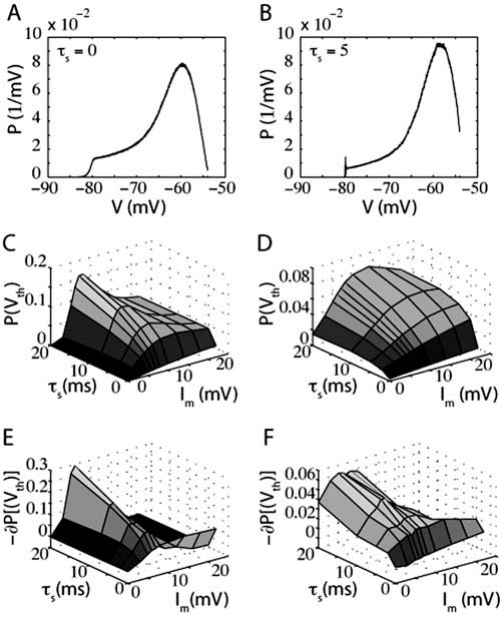
Membrane potential profile of a population of LIF neurons within a finite interval of time. A and B) Membrane potential probability distributions with (A) τ_s_ = 0 ms or (B) τ_s_ = 5 ms. I_m_ was adjusted to that the overall firing rate was 20 Hz. The variance (σ) of the noise was 640 mV^2^-ms. In (A), the nonzero value of P(V) at V = V_th_ arises from the finite time steps that we use by necessity in our simulations. C and D) The value of the probability distribution at spike threshold, P(V_th_), as a function of I_m_ and τ_s_ under (C) low noise and (D) high noise conditions. E and F) Absolute value of the first derivative of the probability distribution at threshold, |∂P(V_th_)|. In the low noise regime the variance of the synaptic component was 160 mV^2^-ms and in the high noise regime it was 1440 mV^2^-ms. (Input is given in mV, the resulting membrane potential depolarization).

The top two panels of Figure 1 display the membrane potential distribution for a population of LIF neurons for τ_s_ of 0 ms (Fig. 1A) and 5 ms (Fig. 1B). When τ_s_ is 5 ms, P(V_th_) is greater than zero (Fig. 1B). In fact, P(V_th_) is a monotonically increasing function of τ_s_ that vanishes in the limit of τ_s_→0 (also see Fig. 1C and Fig. 1D). The direct contribution of the mean current to firing rate comes through a coupling with the value of the probability distribution at threshold, P(V_th_,t), in the drift term [19], [28], [39]. As a result of this coupling, a jump in mean input current, δI, causes an instantaneous jump in the firing rate of the LIF model, δ_I_ν,
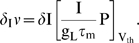
(12)
Figure 1C and 1D demonstrate how P(V_th_) varies as a function of τ_s_ and mean input current for low (Fig. 1C) and high (Fig. 1D) noise conditions. The peaks in P(V_th_) correspond to case where I_m_ is just below the value required to fire the neuron and reflect the hypersensitivity of the firing rate responses at this point to any changes in input. As would be expected, this peak becomes less pronounced and the firing response less sensitive to input parameters as the noise magnitude increases (compare Fig. 1C for lower noise with Fig. 1D for higher noise). In Figure 1 and for the rest of this study, mean input is expressed in mV, the depolarization that results from the input current.

**Figure 2:**
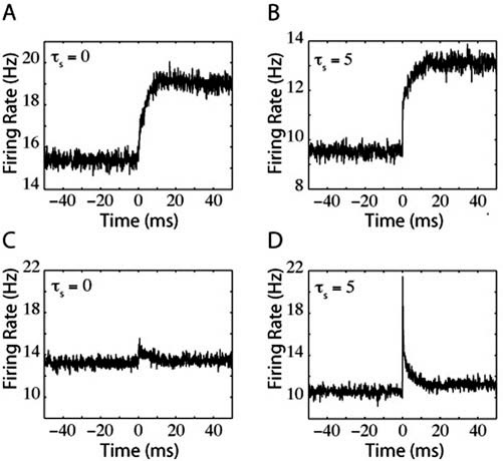
LIF neuron firing rates in response to steps in mean and noise. Each panel is the firing rate of an LIF neuron in response to A) a step in mean input current with τ_s_ = 0 ms, B) a step in mean input current with τ_s_ = 5 ms, C) a step in input current noise with τ_s_ = 0 ms, or D) a step in input current noise with τ_s_ = 5 ms. In (A) there exists a small instantaneous jump that arises because of the finite time steps used in our simulations. For panels (A–C), the variance of the synaptic component was 1440 mV^2^-ms (prior to the input step). In (D), the variance of the synaptic component was 1000 mV^2^-ms and the variance of the external input (prior to the step) was 40 mV^2^-ms.

The trends demonstrated in this figure suggest that a comparably bigger instantaneous response to a mean current jump will be evoked for larger values of τ_s_. In the top two panels of Figure 2, we compare the firing rate of a population of LIF neurons in response to a jump in mean input current for τ_s_ = 0 ms (Fig. 2A) and τ_s_ = 5 ms (Fig. 2B). In Figure 2B, the LIF response to a step in mean input current contains a significant instantaneous component that is not present in Figure 2A.

A jump in mean current pushes the peak of the probability distribution towards spike threshold, instantaneously increasing the probability flow at threshold and inducing an instantaneous jump in firing rate. However, when τ_s_ is very small (for example see Fig. 2A, where τ_s_ = 0 ms), the total firing rate is dominated by the diffusive part of the probability flow (both the diffusive and the drift parts of the probability flow depend on I_m_, see Text S1), and the resulting instantaneous jump in firing rate, δ_I_ν, is negligible compared to the final change in firing rate after the probability distribution reaches its new steady state. Because of the dominance of the diffusive component of probability flow, the firing rate of the LIF model to a small jump in mean current approaches its final steady-state from below (see Fig. 2). However, if the noise level is very low or the synaptic time constant is very large, the response onset will overshoot the final steady-state firing rate (discussed later). At steady-state, the LIF neuron acts like a nonlinear integrator in that its firing rate, ν( I_m_,σ), is primarily determined by the mean input current and only weakly by noise magnitude.

A small jump in noise amplitude, δσ, also results in an instantaneous jump in firing rate, δ_σ_ν,
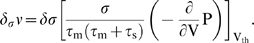
(13)The direct contribution of noise to the firing rate response depends on the first derivative of the probability distribution at threshold, −∂P(V_th_) as it appears in the diffusion term of the probability flow (see eq. 11). The behavior of −∂P(V_th_) determines the response to a step in noise [28]. Figures 1E and 1F show how −∂P(V_th_) varies as a function of τ_s_ and I_m_. Comparison of Figures 1E and 1F with Figures 1C and 1D demonstrates that the dependence of −∂P(V_th_) on τ_s_ is more complex than for P(V_th_). As before, the peaks in the plots correspond to the condition in which the neuron is just below firing threshold and extremely sensitive to changes in input. In the sub-threshold regime, increasing τ_s_ causes an increase in the magnitude of −∂P(V_th_). In the superthreshold regime, however, there is a range in which −∂P(V_th_) decreases with increases in τ_s_. This range corresponds to the situation in which the mean current is far above threshold. Because this trend only occurs for a very small set of parameters that do not correspond to a biologically-realistic situation, we did not investigate it further.

Figures 2C and 2D show firing rates in response to a step in noise for relatively noisy conditions near spike-threshold. Because −∂_V_P is coupled to the magnitude of the noise, σ, the response onset to a jump in noise is always associated with an instantaneous increase in firing rate that “overshoots” the final steady-state value. Examples of this “overshoot” behavior can be seen in Figures 2C and 2D. As stated, in this regime increasing τ_s_ always causes an increase in the magnitude of −∂P(V_th_), enhancing the magnitude of the overshoot. The increase in noise magnitude eventually acts to flatten the probability distribution, decreasing the absolute value of −∂_V_P at firing threshold. The net increase in steady-state firing may thus be relatively small.

Our results demonstrate that sudden small changes in input current will evoke different firing rate changes, δ_I_ν and δ_σ_ν, depending on whether the change is in the mean, δI, or the variance, δσ. Although in this study we focus on using equations (12) and (13) to connect jumps in input to the firing rate behavior during response onset, these equations hold for other patterns of time-varying input.

#### QIF and EIF integrate-and-fire model response onset

**Figure 3:**
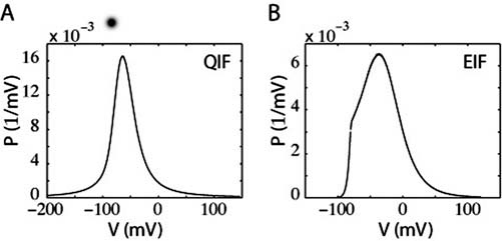
Membrane potential distributions of QIF and EIF models. A) Probability distribution of the QIF model membrane potential. B) Probability distribution of the EIF model. For both panels, τ_s_ = 0 ms and the variance of synaptic component was 9000 mV^2^-ms, resulting in an average firing rate of 20 Hz.

**Figure 4:**
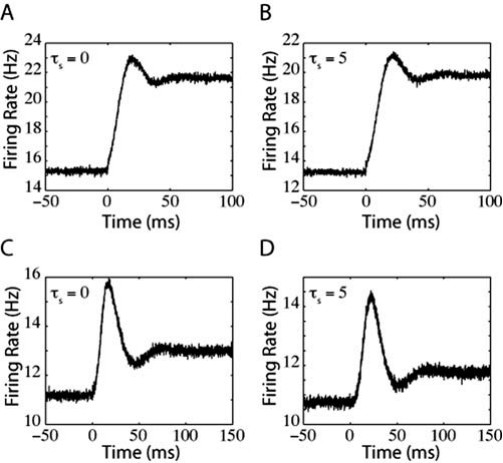
QIF model firing rates in response to a jump in mean input current or noise. A) Response to a step of mean input current with τ_s_ = 0 ms. B) Response to a current step with τ_s_ = 5 ms. C) Response to a step in noise for τ_s_ = 0 ms. D) Response to a step in noise for τ_s_ = 5 ms. For (A) and (B), the variance of synaptic component was 36000 mV^2^-ms. For (C) and (D), the variance of synaptic component (prior to the noise step) was 4000 mV^2^-ms.

Whereas the firing rate of the LIF model is equal to the probability flow at firing threshold, the mean firing rate of the QIF model is equal to the probability flow at infinity. The probability distribution of the QIF model is shown in Figure 3A (Figure 3B is the probability distribution of the EIF model, discussed next). As described previously, the probability distribution in this model decays exponentially for depolarized values of membrane potential. As a result, only a negligible fraction of the population is near threshold at any given time. (Note that for both QIF and EIF models, the dynamics are such that neurons approach infinity extremely rapidly. Thus firing can occur even though such a small population of neurons are near threshold). In the large V limit, there is no direct coupling between the probability flow and the mean current or noise,
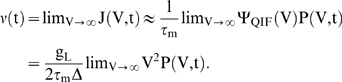
(14)As a result, the QIF model response to a jump in either mean input current or noise does not contain an instantaneous component. Figure 4 displays examples of QIF responses to jumps in either mean (Fig. 4A and B) or noise (Fig. 4C and D). As for the LIF neuron, the level of noise and the size of the synaptic time constant affect whether the firing rate smoothly approaches the final steady-state value from below or overshoots its value after a step of input current. Also as the LIF neuron, a step in noise results in a transient overshoot of the final firing rate, although this overshoot is not instantaneous. The most significant difference between the response onset of the LIF and the QIF model is the lack of an instantaneous component for the QIF response. This difference arises primarily because the QIF model does not include a hard spike threshold.

**Figure 5:**
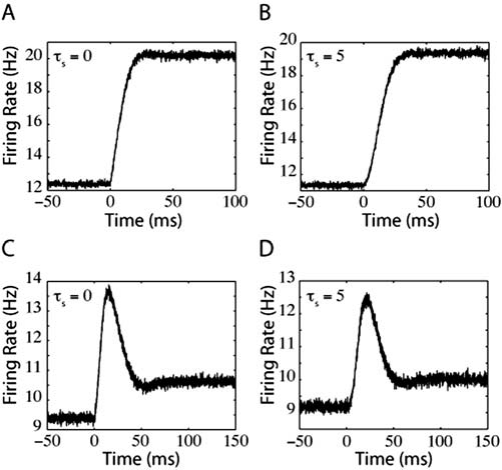
EIF model firing rates in response to a jump in mean input current or noise. A) Response to a current step with τ_s_ = 0 ms. B) Response to a current step with τ_s_ = 5 ms. C) Response to a step in noise for τ_s_ = 0 ms. D) Response to a step in noise for τ_s_ = 5 ms. As in Fig. 4, for panels (A) and (B), the variance of synaptic component was 36000 mV^2^-ms. For (C) and (D), the variance of the synaptic component (prior to the noise step) was 4000 mV^2^-ms.

Similar to the QIF model, the response onset of the EIF model also does not contain an instantaneous component. The probability distribution of the EIF model is given in Figure 3B. The EIF probability distribution function dies off as an exponential of an exponential at depolarized values of V. As with the QIF model, the firing rate depends only indirectly on input current variables because of the low probability distribution near spike threshold. As a result, there is no instantaneous component in the response onset to step functions of either mean current (Fig. 5, A and B) or noise (Fig. 5, C and D). Also as the QIF model, the presence of an overshoot of the final steady-state firing rate depends on the level of noise and size of synaptic time constant. Such an overshoot is clearly visible in Figure 5C and 5D. There is virtually no overshoot visible in Figures 5A and 5B because the overshoot of the EIF model dies off faster than that of the QIF model for the same level of noise variance (see Decaying Response to Step Function). In the limit of Δ→0, the EIF model turns into the LIF model, and in this limit, the overshooting component becomes equivalent to the instantaneous response onset of the LIF model.

#### Conductance-based models response onset

Previous work and the results discussed in the previous sections show that the action potential threshold mechanism appears to play a critical role in the response onset [27]. For this reason we examined the response onset in a more biologically-realistic conductance-based single-compartment neuron model [36] (also see Methods). In the absence of noise, the dynamics of the conductance-based model (when it is firing) forces it through a closed loop trajectory (due to the existence of a limit cycle attractor) in its D-dimensional configuration space. With the addition of noise, this trajectory widens to a D-dimensional closed tube [15], although for a realistic noise magnitude, the trajectory is almost a 1-dimensional loop in configuration space. Because of this, we may reduce the probability distribution to a two-dimensional subspace of configuration space and still access sufficient information to understand the behavior of the model. We have chosen P(V,n) for this purpose, where n is the potassium channel gating variable (see Methods).

**Figure 6:**
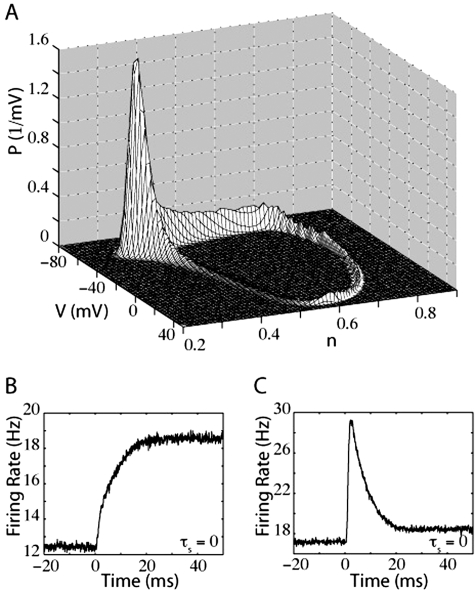
Membrane potential distribution of the conductance-based model. A) Probability distribution of the conductance-based model, plotted against membrane potential (V) and the potassium gating variable (n). The variance of the synaptic component was 1000 mV^2^-ms. B) Firing rate of the conductance-based model in response to a step of input current. The synaptic time constant, τ_s_, was 0 ms. The variance of synaptic component was 4000 mV^2^-ms. C) Firing rate of the conductance-based model in response to a step of noise, with τ_s_ = 0 ms. The variance of synaptic component (prior to the noise step) was 2250 mV^2^-ms.

The probability distribution of the conductance-based model, while firing, is plotted in this reduced representation in Figure 6A. The bulk of the probability distribution is located at subthreshold membrane potentials (left-rear in Fig. 6A). During an action potential, the response trajectory travels counter-clockwise in the figure. During the depolarizing phase of an action potential, the neuron travels forward and to the right on the figure, representing depolarization of the membrane potential and activation of voltage-gated potassium channels. During the repolarization phase of the action potential, the neuron hyperpolarizes and potassium channel activation decreases as the neuron travels into the left-rear of the figure.

Because the spike-generation mechanism of the conductance-based model is very fast relative to the temporal dynamics of the subthreshold membrane potential, only a small subpopulation of neurons exists in the action potential regime at any time, including the regime near spike-detection threshold. As with the QIF and EIF models, the response onset following a step in mean or noise input does not have an instantaneous component (see Fig. 6B), although (also as with QIF and EIF model neurons), a step in noise causes a sudden increase in probability flow towards higher membrane potentials that results in a fast transient (see Fig. 6C). However, unlike the LIF model, this transient response, although rapid, is not instantaneous. The rise time of the membrane potential during the upward phase of the action potential is very brief relative to the repolarization time. This can be seen directly by inspecting the voltage trace of an individual spike (not shown) or by comparing the size of the probability distribution during the upward phase of the action potential (foreground) with the probability distribution during the downward phase (background). The fast rise time is on the order of a few milliseconds, which is exactly the time to peak in the firing rate transient that occurs after the jump in noise.

### Decaying Response to Step Function

A general feature of firing responses to step functions in either mean or variance displayed by all models in this study is a decaying oscillation towards the new firing rate value. Any jump in input creates a disparity between the probability distribution profile (the steady-state solution immediately before the jump) and the new steady-state solution. For a population of neurons, this initial imbalance has a synchronizing effect and creates oscillations in the firing rate across the population [16]. This synchronized firing arises because of the simultaneous change in input across the population and not through any coupling between neurons in the population. The period of the oscillations is determined by the final firing rate because it is directly related to the average interspike interval of the firing response of any one neuron in the population.

**Figure 7:**
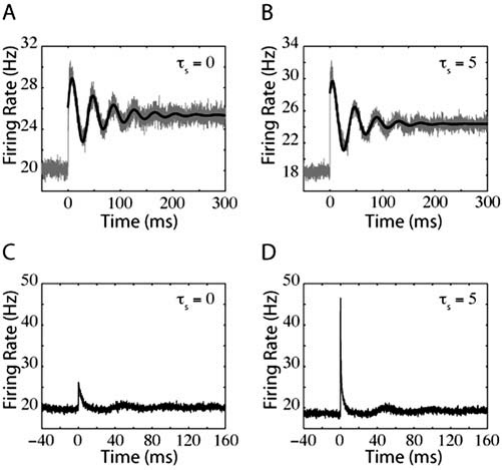
LIF oscillating response to jumps in mean input current and noise. For the top panels, the jumps in firing rate were driven by steps in mean input current. For the bottom panels, the model neurons are responding to steps in noise. In panels (A) and (C), τ_s_ = 0 ms and the variance of synaptic component was 10 mV^2^-ms. In panels (B) and (D), τ_s_ = 5 ms. Prior to the step in noise, the variance of synaptic component was 90 mV^2^-ms in (C) and 40 mV^2^-ms for the variance in synaptic component and 10 mV^2^-ms for the external input variance in (D).

**Figure 8:**
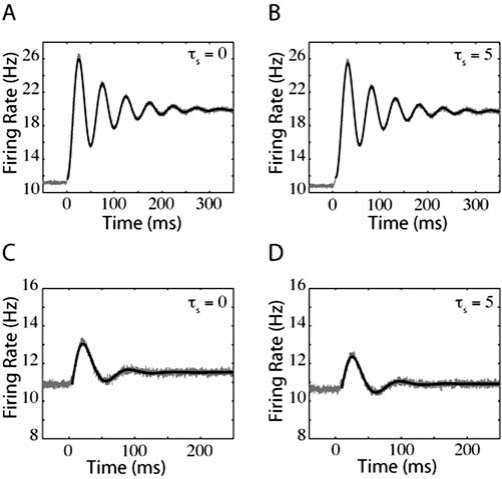
Oscillatory QIF responses to jumps in mean input current and noise. The top panels are QIF firing rates in response to jumps in mean input current and the bottom panels are QIF firing rates in response to jumps in noise. For panels (A) and (C), τ_s_ = 0 ms. For panels (B) and (D), τ_s_ = 5 ms. The variance in synaptic component was 4000 mV^2^-ms for (A) and (B), or 2250 mV^2^-ms prior to the step in noise for (C) and (D).

**Figure 9:**
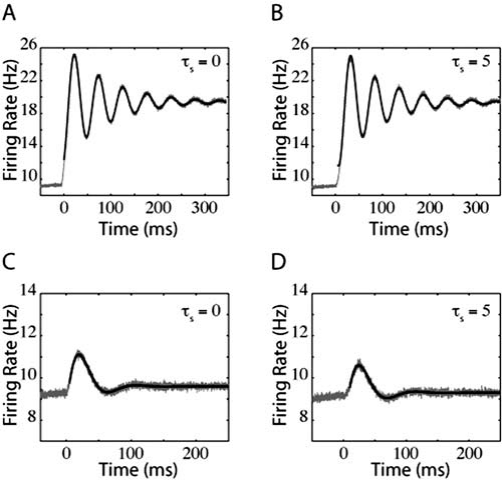
EIF oscillatory responses to jumps in mean input current and noise. The top panels of EIF firing rates in response to jumps in mean input current and the bottom panels are EIF firing rates in response to jumps in noise. As in figures 7 and 8, for panels (A) and (C), τ_s_ = 0 ms, and for panels (B) and (D), τ_s_ = 5 ms. As in Fig. 8, noise variance was 4000 mV^2^-ms for (A) and (B), or 2250 mV^2^-ms prior to the step in noise for (C) and (D).

The noisy component of the input current eventually cancels the mismatch between the steady-state probability profiles before and after the input step by allowing the potential distribution to asymptotically approach the new steady-state distribution. The higher the magnitude of the noise, the faster the firing rate relaxes to its final steady firing rate. For relatively small jumps in input current parameters, it is possible to asymptotically fit the firing rate with only one decaying component,
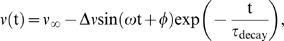
(15)where τ_decay_ describes the time scale of relaxation. The thin black lines in Figures 7, 8, and 9 are the fits of such oscillating, decaying functions. These figures demonstrate the firing-rate response (grey lines) of the LIF, QIF, and EIF models, respectively, to jumps in input with lower levels of noise than in Figures 2, 4, and 5 which enhances the oscillations. Because of the relatively low levels of noise, the firing rate responses in Figures 7, 8, and 9 overshoot the final steady-state firing rate.

The firing-rate dynamics of our models can be understood by studying the Fokker-Planck equation that governs the dynamics of the probability distribution, P(V,t). The Fokker-Planck operator L_FP_ explicitly depends on the input-current mean and variance. The spectrum of the FP operator, λ_0_(t), λ_1_(t), … , defines a hierarchy of time scales. For time scales Δt that are much larger than 1/|Re(λ_2_(t))| the dynamics of FP equation can be replaced by a simple oscillator. In particular, the firing rate of our noisy population is the real part of ν(t) in the following first-order differential equation
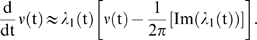
(16)The asymptotically decaying oscillatory behavior after a jump in input parameters is a general solution to this equation. The final firing rate is proportional to the imaginary part of λ_1_(t) while the decay time constant is related to the inverse of the real part of λ_1_(t).

**Figure 10:**
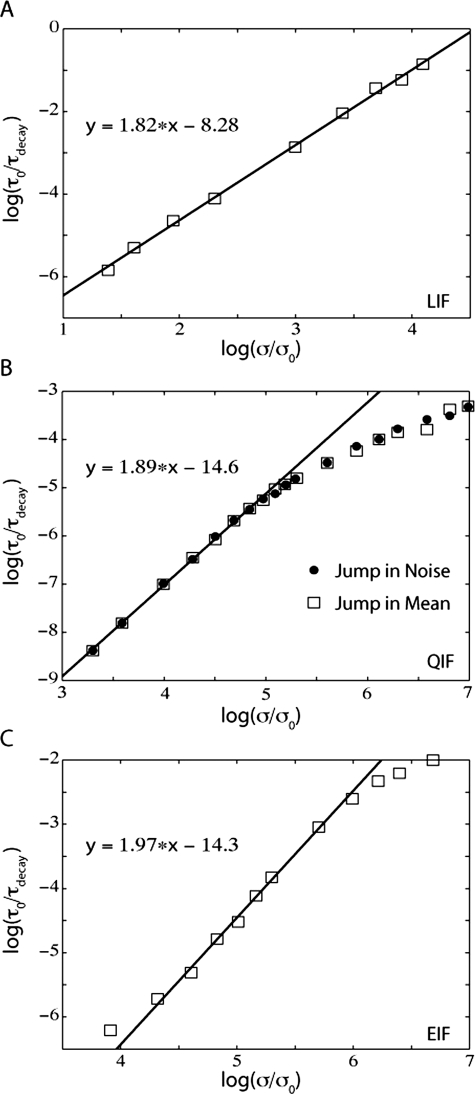
Oscillation decay time constants for the integrate-and-fire models vary as power functions of noise. LIF τ_decay_ (A), QIF τ_decay_ (B), and EIF τ_decay_ (C) are given as functions of final noise magnitude (noise level after the jump in noise). For the QIF model (B), the decay time constants measured from responses to a jump in mean are given by empty squares and the decay time constant measured from responses to jumps in noise are given by filled circles. τ_0_ = 1 ms and σ_0_^2^ = 0.1 mV^2^-ms.

Interestingly, the relationship between τ_decay_ and noise magnitude follows a power law for a large range of parameters
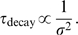
(17)As shown in Figure 10, this power-law relation holds across all IF models in the limit of small noise magnitudes. This relationship holds whether the input jump is in mean (open squares in Figures 10A, 10B, and 10C are for the LIF, QIF, and EIF models, respectively) or noise (demonstrated for the QIF model in Fig. 10B, filled circles). The relationship between τ_decay_ and noise magnitude can be understood through a perturbative calculation of the first non-zero eigenvalue of the Fokker-Planck equation for small magnitudes of noise. We can break the Fokker-Planck operator L_FP_(I,σ) into a noise-independent component and a noise-dependent component, i.e. L = L_0_+σ^2^L_1_. The appearance of the multiplicative σ^2^ term causes the perturbative expansion of all eigenvalues in increasing powers of σ^2^. In particular, the real part of the first non-zero eigenvalue is dominated by a σ^2^ term in the small noise limit.
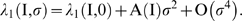
(18)In addition, direct numerical analysis of the real part of the first non-zero eigenvalue as a function of noise magnitude confirms the quadratic dependence. The dependence of the real part of the first non-zero eigenvalue of the Fokker-Planck equation on noise magnitude for the super-threshold QIF model is quadratic (not shown). The relation τ_decay_ = 1/|Re(λ_2_)| that was introduced in the above equation can be used to explain the power-law dependence of τ_decay_ on σ. The analysis for the QIF model is drastically simplified because the whole parameter space (I,σ) can be mapped by scaling time and membrane potential to three 1-dimensional subspaces (I = −1,0,+1,σ) [40]. The sub and supra-threshold regions are reduced to the I = −1,+1 subspaces, and the case with fine-tuned balanced input is the I = 0 subspace.

When the jump in input is large relative to the pre-jump value, the initial response overshoots the expected decaying oscillation for both QIF and EIF models (for examples, see Figs. 8 and 9). This overshoot occurs because the higher eigenvalues in the spectrum of Fokker-Planck operator become relevant in the firing-rate calculation. This overshoot can also be thought of as analogous to the instantaneous jump observed in the LIF model, which can be recovered from the EIF model in the limit of Δ→0. This explains why the overshoot is more significant in noise jumps (see Fig. 4C, Fig. 4D, Fig. 5C, and Fig. 5D). After a step increase in noise, the initial membrane potential probability distribution widens. The coupling between noise magnitude and the slope of the membrane potential probability distribution causes a sudden increase in probability flow towards higher potentials, pulling more neurons to the spike generation potential. Because the spike generation mechanism in QIF and EIF models is not instantaneous, the sudden increase in probability flow in the finite V region appears as a delayed overshoot. This delay corresponds to the time neurons take to reach infinity (thus firing an action potential) from a membrane potential near the peak of the probability distribution. Due to the V↔−V symmetry in Ψ(V) of the QIF model, this time is approximately equal to half of the average inter-spike time interval.

Because the quadratic term in the Ψ(V) function dominates spike generation in the QIF and EIF models, differentiating between their firing responses can be difficult. For each model, we adjusted Δ to set the Ψ(V) functions of the QIF and EIF models to have the same radius of curvature at their minimum (see Methods). As a result, the responses of both models are quite similar. A comparison between Figure 4 and Figure 5, illustrating QIF and EIF model responses under the influence of equal amounts of noise, however, indicates that the EIF model dynamics is more sensitive to noise. For example, the decaying response of the EIF model in Figure 5 “forgets” the step in input much sooner than the QIF model in Figure 4. We believe that this difference arises because of the slower refractory period of the EIF model (the linear vs. quadratic dependence of Ψ(V) on V for large negative V and results in smaller values of Ψ(V)).

#### Conductance-based models decaying response

**Figure 11:**
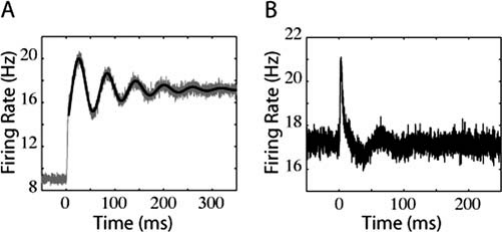
Oscillating responses of the conductance-based model under lower noise conditions. A) Firing rate of the conductance-based model in response to a step of input current. The synaptic time constant, τ_s_, was 0 ms. The variance of synaptic component was 722.5 mV^2^-ms. B) Firing rate of the conductance-based model in response to a step of noise, with τ_s_ = 0 ms. The variance of synaptic component was 160 mV^2^-ms.

We also studied the responses of the conductance-based neuron to sudden jumps in mean and noise. The initial response to a jump in either mean or noise begins with a sharp onset (discussed earlier) followed by a decaying oscillation, as shown in Figure 11 (grey lines). Again, the thin black lines are fits of an exponentially decaying sinusoidal function. Just as for the IF models, the period of oscillation and decay rate depend on the final firing rate and input variance after the jump in input. The sharp onset, especially in the case of a noise jump, is a result of having a large population of neurons very near the potential at which the action potential is triggered. The time lag between action potential initiation and detection is reflected in the presence of the sharp, though not instantaneous, onset.

## Discussion

We have studied the temporal dynamics of the firing rate response of integrate-and-fire and conductance-based models to rapid changes in mean or noise. For analysis purposes, we divided the time course of the population response into two regimes. The initial response, “response onset”, indicates how fast the population reacts to a change in its input. The asymptotic behavior of the response as it approaches its final steady-state value, referred to in this paper as the “decaying response”, is described by a characteristic time scale, τ_decay_. Any signals with time scales slower than τ_decay_ will be reflected in population firing rate with little distortion.

The temporal firing rate response of an integrate-and-fire model can be predicted based on the characteristics of the membrane potential probability distribution near threshold and the coupling between the probability flow and the input current (for a review see [41]). In this study we focused specifically on the leaky integrate-and-fire model, the quadratic integrate-and-fire model, and the exponential integrate-and-fire model because of their mathematical tractability. For the LIF model the response onset to a step in mean current appears as an instantaneous jump in firing rate for non-zero τ_s_. Because this instantaneous component arises from a non-zero value of the probability distribution at spike threshold, it is absent when τ_s_ equals zero. For a jump in noise, the LIF response onset always contains an instantaneous component and overshoots the final steady-state firing rate. Within the range of firing rates that we studied, the size of the response onset increases for larger synaptic time constants due to increases in the values of both the probability distribution of the membrane potential and its derivative at spike threshold for larger values of the synaptic time constant.

The firing rates of the QIF, EIF, and conductance-based models, on the other hand, change smoothly, even in response to an instantaneous increase in input current. This property is due to the fast decay of the membrane potential distribution at relatively depolarized (and thus close to spike detection threshold) values. Silberberg et al have previously shown that living neurons also respond to a step in noise with a rapid rise in firing rate [28], similar to the behavior of the conductance-based model shown here. For the EIF and QIF models, Fourcaud-Trocmé and Brunel [27] have found that, in the low noise regime, the slope of the firing rate increases during the brief time interval immediately after a sudden jump in mean is slower than the corresponding increase for a jump in noise, and that the reverse is true in high noise conditions. With close inspection, the time interval with an approximate linear rise in firing rates can be seen in Figures 4 and 5.

All IF model responses to relatively small jumps in mean current or noise in the asymptotic region can be fit to exponentially decaying oscillations for small τ_s_ (i.e. τ_s_≪τ_m_). The decay time constant has a power law dependence on the magnitude of the background noise. We focused on the firing response of various IF neurons at t = 0 (response onset), and at t→∞ (decaying response). For the parameter range we studied, QIF and EIF responses to large steps in mean or noise cannot be fit to a simple decaying oscillation due to the importance of more rapidly decaying modes. For small input jumps, the fit matches quite well, although there are overshoots near t = 0. Also, the responses tend to decay faster and appear sharper after a jump in noise than a jump in mean. This sharpening of the response is due to the increased level of noise. As mentioned earlier, overshoots arise through the contributions of higher harmonics (eigenfunctions). The expansion coefficients of these rapidly decaying modes (a_n_ in equation 9 of Text S1) decrease with a power law as a function of n for large n, i.e. lim_n→∞_ a_n_∝ n^−β^. This relation is due to the existence of ∂^2^/∂V^2^ (the curvature of a function) in the Fokker-Planck operator, L_FP_, which makes higher eigenfunctions more oscillatory functions of V. The summation of these faster modes adds up to the sharper appearance of oscillation just after the jump.

An increase in noise reduces the decay time constant, allowing the firing rate to more faithfully follow the input current. This process is much like “dithering”, a technique used to minimize artifacts in signal transmission. We can define the error in transmission of a jump in mean or noise as the average in a time window T of the difference between 1 and relative final firing rate ν(t)/ν(t_∞_). This parameter was named the “dissimilarity” parameter for the more general case of an arbitrary input [42]. At this level, T is an arbitrary parameter but it may be thought of as the characteristic time scale of an input. There are two major contributing factors to the dissimilarity between the input and the output firing rate, the *“systematic error”*, arising from the oscillatory behavior (synchronization) displayed by all IF models converging towards their final firing rate, and the *“random error”*, the random component of the response of each neuron. Although the random error decreases in the large N (the number of network neurons) limit, the systematic error persists even as N→∞. Increasing the input noise causes firing rates to converge to their final values more quickly and decreases the systematic error, although at the same time it also increases the random error. The competition between these effects of σ on the dissimilarity parameter indicate that, regardless of the model under consideration, there exists a non-zero level of noise, σ_opt_, that optimizes signal transmission.

The optimal value of noise will depend in part on the time scale of the encoded signal. Any input signal can be approximated by a piece-wise constant function with jumping period of T. The variables σ_opt_ and T are dependent since T appears in a factor of 1-exp(T/τ_decay_) in the dissimilarity parameter if eq. (10) approximates the firing rate well at all times. The weak dependence of σ_opt_ on T in the large noise limit can be the basis for a robust mechanism of fast and faithful signal transmission. In contrast, in the small noise regime T and σ_opt_ are strongly correlated and optimizing signal transmission requires that the system adjust the magnitude of noise.

## Supporting Information

Text S1 (0.10 MB DOC)
