# Supplementary material for: Transient Responses to Rapid Changes in Mean and Variance in Spiking Models
Source: PLoS One. 2008 Nov 21;3(11):e3786. doi: 10.1371/journal.pone.0003786 (PMC2582948; doi:10.1371/journal.pone.0003786)
Supplement: Text S1 — (0.10 MB DOC) [file pone.0003786.s001.doc]

Transient responses to rapid changes in mean and variance in spiking models

Peyman Khorsand and Frances Chance

**Supplemental Information**

**Fokker-Planck Equation:**

Explicit integration of the membrane potential dynamical equation requires knowledge of the total current I(t), but because of the noise component of I_syn_,

|  |  |  |
| --- | --- | --- |

this cannot be known explicitly. Instead, we define a probability distribution, P(V,I,t), as a function of the membrane potential and the input current. The probability distribution may be visualized as a cloud of many individual neurons with many individual trajectories in configuration space (the space spanning all possible values of the independent variables used in our study). P(V,I,t)ΔVΔI therefore, describes the ratio of the expected number of neurons and the total number of neurons (or, if we concentrate on one neuron, the probability of finding that particular neuron) in the ranges of [V, V+ ΔV] and [I, I+ ΔI] at time t. We study the dynamics of the probability distribution in (V,I) configuration space.

The net flux of the trajectories passing through any surface in the (V,I) space is described by the component of the probability flow vector, **J**, perpendicular to that surface. Although by some conventions **J** is referred to as the probability current vector, we chose “probability flow” to avoid confusion with ionic currents present in the neuronal membrane. In a 2-dimensional noisy dynamical system, such as the one at hand, P(V,I,t) satisfies a 2+1-dimensional partial differential equation known as the Fokker-Planck (FP) equation. The FP equation is effectively a conservation/continuity equation for the probability distribution. It connects any inhomogeneity of the probability flow, **J**(V,I,t), in configuration space to the change in local probability distribution over time:

|  |  |  |
| --- | --- | --- |

In the above equation, J_V_(V,I,t) and J_I_(V,I,t) are different components of the probability flow vector. Each component represents the projection of the probability flow vector in that direction. The probability flow vector may be broken into two different pieces, a “drift” term and a “diffusion” term. The drift term specifies the deterministic part of the collective motion of the neurons in configuration space, and can be described as the product of a velocity-like factor and the probability density, P. The diffusion part of **J**, on the other hand, originates from the noisy indeterministic dynamics of the system, and acts to diminish any peak in the probability distribution function.

In Ito's interpretation of the stochastic differential equation for an integrate-and-fire neuron, the drift terms of the different components are:

|  |  |  |
| --- | --- | --- |

|  |  |  |
| --- | --- | --- |

Here τ_m_ is the membrane time constant, equal to the membrane capacitance divided by the membrane conductance, g_L_. The diffusion terms are:

|  |  |  |
| --- | --- | --- |

|  | , |  |
| --- | --- | --- |

In the limit of τ_s_ → 0, the synaptic current can be found algebraically, I_syn_(t) = I_m_(t) + σ(t)η(t). For the LIF model, equations (11) and (12) from the main text can be combined into one equation and the configuration space becomes 1-dimensional. During this dimensional reduction process, the information about the boundary condition is lost. Finding the finite value of P(V_th_,t) for τ_s_ > 0 is a difficult and unsolved problem and in the main text the values are found through simulations.

The boundary conditions imposed on the Fokker-Planck equation, as well as the (V) term, are model-dependent. For each model, the firing rate is directly related to the integral of the normal outward probability flow across the boundary determined by spike-threshold. For analysis, we try to symbolically separate time dependence from the dependences on other variables. The FP equation then takes the form:

|  |  |  |
| --- | --- | --- |

Where $\hat{L}_{\text{FP}}$ is the so-called Fokker-Planck operator.

***Spectral Analysis of Fokker-Planck Equation:***

For the case of an IF neuron with constant mean, constant noise magnitude, and τ_s_ = 0 , the FP operator is time-independent, and it is useful to look at its spectrum,

|  | , |  |
| --- | --- | --- |

A general solution to the initial-value problem can be found if both the spectrum and eigenfunctions of the FP operator are known

|  | . |  |
| --- | --- | --- |

Each a_n_ is a constant determined by the initial probability distribution [S1]. In general, the time-independent FP equation always has a steady-state solution that corresponds to λ_0_ = 0, with all the other eigenvalues having negative real parts to satisfy stability requirements. After sorting the eigenvalues by their real part, 0 = Re(λ_0_) ≥ Re(λ_1_) ≥ Re(λ_2_) ≥…, the probability distribution can be asymptotically approximated by keeping only the first two terms in the above equation,

|  | . |  |
| --- | --- | --- |

This approximation works best if |Re(λ_1_)| << |Re(λ_2_)|. The firing rate of the integrate-and-fire neuron is directly related to the probability flow and consequently to the probability distribution, and may be approximated (by adiabatic approximation) as the imaginary part of the first nonzero eigenvalue of the FP equation, ν(t) = |Im(λ_1_(t))|/(2π). This approximation holds as long as the input current does not vary significantly for time scales smaller than ~1/|Re(λ_1_)|.

A better approximation uses information in both the real and imaginary parts of λ_1_,

|  |  |  |
| --- | --- | --- |

The above equation indicates that the firing rate decays toward its expected value, |Im(λ_1_(t))|/(2π), with a time constant set by the inverse of the real part of λ_1_(t). The time dependence of the input current enters the above equation through the time dependence of the variables involved in the eigenvalue λ_1_(t) = λ_1_(I_m_(t),(t)). This approximation is valid provided that the input does not vary significantly at time scales on the order of 1/|Re(λ_2_)|, and suggests that the firing rate will follow an asymptotically decaying, oscillatory behavior to its new value after a sudden jump in mean current or noise magnitude (for example, see Figures 7, 8, and 9),

|  |  |  |
| --- | --- | --- |

In this approximation, the imaginary component of the first nonzero FP eigenvalue, ω = |Im(λ_1_(t))|, determines the frequency of oscillation. The real component, on the other hand, controls the rate of decay according to τ_decay_ = 1/|Re(λ_1_(t))|.

**References**

S1. Mattia M, Del Giudice P (2002) Population dynamics of interacting spiking neurons. Phys Rev E 66: 051917.
